# Investigating regional-specific gut microbial distribution: an uncharted territory in disease therapeutics

**DOI:** 10.1093/procel/pwae058

**Published:** 2024-10-29

**Authors:** Junliang Kuang, Xiaojiao Zheng, Wei Jia

**Affiliations:** Center for Translational Medicine and Shanghai Key Laboratory of Diabetes Mellitus, Shanghai Sixth People’s Hospital Affiliated to Shanghai Jiao Tong University School of Medicine, Shanghai 200030, China; Center for Translational Medicine and Shanghai Key Laboratory of Diabetes Mellitus, Shanghai Sixth People’s Hospital Affiliated to Shanghai Jiao Tong University School of Medicine, Shanghai 200030, China; Center for Translational Medicine and Shanghai Key Laboratory of Diabetes Mellitus, Shanghai Sixth People’s Hospital Affiliated to Shanghai Jiao Tong University School of Medicine, Shanghai 200030, China; Department of Pharmacology and Pharmacy, University of Hong Kong, Hong Kong SAR, China

The gastrointestinal (GI) tract is crucial for nutrient digestion and absorption. Longitudinally segmented into the mouth, esophagus, stomach, duodenum, jejunum, ileum, cecum, and colon, each region plays a critical role in these processes and hosts trillions of symbiotic microbiota with diverse functions (Donaldson et al., 2016). Moreover, as the body’s largest endocrine organ, the GI tract orchestrates host metabolic regulation via a complex crosstalk between the host and microbiota, mediated by gut hormones, bioactive peptides, and microbiota-modified metabolites (Ahlman and Nilsson, 2001; Donaldson et al., 2016).

Conventionally, it was believed that only a limited number of microbiota in the oral cavity could move beyond their original location and colonize other regions of the GI tract, potentially causing metabolic disorders. However, recent studies have increasingly demonstrated that microbes residing in the oral cavity are significantly linked to systemic diseases (Baker et al., 2024; Peng et al., 2022), including metabolic and GI disorders (Read et al., 2021). Interestingly, some studies have found that the atypical colonization of oral microbes in the lower intestinal tract could contribute to GI immune (Atarashi et al., 2017) and glycolipid metabolic disorders (Kunath et al., 2022; Vonaesch et al., 2022). While the driving mechanism behind microbial migration warrants further investigation, these findings underscore the fact that microbial dislocation in the gut occurs much more frequently than previously anticipated.

The small intestine, including the duodenum, jejunum, and ileum, serves as the primary site for nutrient processing, with each segment displaying distinct metabolic patterns. Physiological factors, including enzymes such as pepsin and gastric lipase from the stomach, bile acids (BAs) from the liver, and trypsin, amylase, and carboxypeptidase from the pancreas, modulate the functionality of these segments (Peters, 1970; Treherne, 1967). Consequently, the upper intestine’s local microenvironment influences the survival, colonization, and proliferation of microbiota, resulting in a lower bacterial density due to factors such as oxygen presence, rapid transit time, variable pH, and enzymatic activity (Kastl et al., 2020; Martinez-Guryn et al., 2018). Notably, aging significantly alters the gut microbiota signatures of the small intestine, potentially becoming microbial hallmarks of aging in the future (Leite et al., 2021). On the other hand, the large intestine, comprising the cecum and colon, harbors the most abundant bacteria due to its thicker mucus layer and favorable conditions for microbial growth (Evans et al., 1988; Marteau et al., 2001; Pereira and Berry, 2017). The variations in physiological characteristics (such as epithelial structure and intestinal motility), microenvironments (aerobic/anaerobic), and pH levels along the GI tract contribute to the biogeographic distribution of the microbiota, which has already been systematically reviewed before (de Vos et al., 2022; Martinez-Guryn et al., 2019).

Recent multi-omics studies have revealed significant differences in microbial composition, host proteins, metabolome, and phages among different regions of the GI tract (Folz et al., 2023; Shalon et al., 2023; She et al., 2024). These findings underscore the spatial heterogeneity of the microbiota, metabolites, and immune response across different segments of the intestine. Consequently, the displacement of bacteria from their native niches can occur in response to diverse physiological and pathological stimuli. Disruption of the microbiota–intestinal immune homeostasis ultimately leads to systemic metabolic dysregulation and contributes to metabolic and GI diseases. Manipulating gut microbiota redistribution may offer a novel therapeutic approach for metabolic diseases.

## The longitudinal distribution and functionality signatures of microbiota and metabolites along the gastrointestinal tract

**Figure 1.**
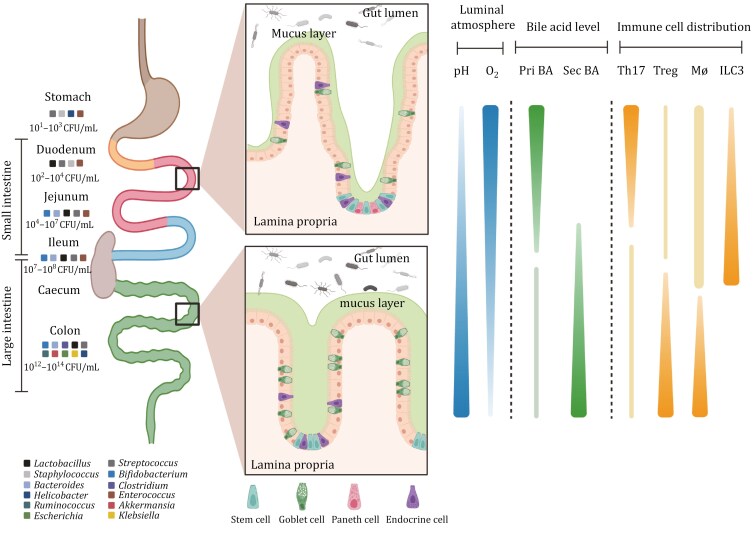
The longitudinal microbial distribution and other physiological characteristics of GI tract. The left side illustrates the bacterial composition and colony-forming units (CFU/mL) in different regions of the gut, from the stomach to the colon. The middle insets provide detailed views of the epithelial structure in the small intestine and colon, showing key cell types such as stem cells, goblet cells, Paneth cells, and endocrine cells, along with the lamina propria and the mucus layer. The right side illustrates the luminal atmosphere parameters (pH, oxygen levels), bile acid concentration (primary and secondary bile acids), and the distribution of key immune cell types along the GI tract. The gradients represent variations in these factors across different regions of the intestine. Pri BA, primary bile acid; Sec BA, secondary bile acid; Th17, T helper 17 cells; Treg, regulatory T cells; Mø, macrophages; ILC3, type 3 innate lymphoid cells.

The gastrointestinal tract include longitudinal segments (i.e., stomach, duodenum, jejunum, ileum, cecum, and colon), and each region play critical roles in digestion and absorption processes of dietary nutrients, meanwhile, as the largest endocrine organ regulating host metabolism through various gut hormones and bioactive peptides (Ahlman and Nilsson, 2001; Donaldson et al., 2016) (Fig. 1). The small intestine which could be further divided into three segments (duodenum, jejunum, and ileum, each with distinct metabolic patterns), is responsible for the digestion and absorption of dietary nutrients. These physiologic functions were affected by many physiological factors, such as pepsin and gastric lipase from the stomach, bile acids (BAs) from the liver, trypsin, amylase, and carboxypeptidase secreted from the pancreas (Peters, 1970; Treherne, 1967). The microenvironment of each gut segment affects the survival, colonization, and amplification of microbial organisms, the upper small intestine harbors a lower abundance of bacteria as the O_2_ contents, transit time, pH levels, and presence of digestive enzymes (Kastl et al., 2020; Martinez-Guryn et al., 2018). Apart from these intestinal physiologic factors, a recent study found that natural aging significantly altered the gut microbiota signatures of the small intestine (Leite et al., 2021), which may become the hallmarks of aging from the microbial perspective in the future. Interestingly, host genetic variations attributed by the individual diversity in immune-related pathways could greatly impact the microbial composition in different human body sites (Blekhman et al., 2015).

The large intestine which included the cecum and colon, harbors the most abundant bacteria owing to their thicker mucus layer and luminal microenvironment suitable for microbial growth (Evans et al., 1988; Marteau et al., 2001; Pereira and Berry, 2017). Moreover, the characteristics of bacteria along the gastrointestinal tract have been reviewed in some papers (Donaldson et al., 2016; Martinez-Guryn et al., 2019). The physiologic properties, colonization preference of microbes, and gut luminal microenvironment determine the longitudinal distribution of gut microbiota and its related metabolites along the gastrointestinal tract (Table 1). Therefore, when bacteria did not colonize in their original location, after exposure to various external physiological or pathological stimuli, it may lead to the occurrence of metabolic or gastrointestinal diseases. We reckon that the longitudinal distribution of gut microbiota in each intestinal segment is disturbed in metabolic and gastrointestinal diseases, which contributes to the onset and progression of the diseases.

**Table 1.** The relationship between regional-specific microbiota, metabolites, and diseases.

Metabolite	Key microbial species	GI tract location	Disease
	*Bacteroidia*, *Gammaproteobacteria, Clostridia*, *Bacilli*, *Alphaproteobacteria*, *Actinobacteria*, *Campylobacteria*, *Fusobacteria*, *Negativicutes*, *Erysipelotrichia*, *Lentisphaeria*, *Coriobacteriia*, *Mollicutes*, *Chlamydiae*, *Deltaproteobacteria*, *Verrucomicrobiae* and *Saccharimonadia*	Duodenum	Hyperglycemia (Darra et al., 2023)
	*Streptococcus*, *Prevotella lineages*, *Fusobacteria*, *Veillonella*	Duodenum	Functional dyspepsia, gastric emptying (Shanahan et al., 2023)
	*Escherichia coli*, *Prevotella salivae*, *Neisseria*	Duodenum	Celiac disease (Constante et al., 2022)
Bile acids			
DCA, LCA	*Clostridium*, *Lactobacillus*, *Bifidobacterium*, *Bacteroides*	Distal small intestine and colon	CRC (Gérard, 2013; Ridlon et al., 2016b)
Iso-DCA	*Eubacterium lentum*, *Clostridium perfringens*, *Ruminococcus gnavus*	Distal small intestine and colon (Wahlström et al., 2016)	
Allo-bile acids	*Eubacterium*	Distal small intestine and colon	HCC (El-Mir et al., 2001)
UDCA	*Clostridium*	Distal small intestine and colon	Cholesterol gallstone, PBC and PSC (Ridlon and Bajaj, 2015)
HDCA	*Unidentified Gram-positive rod*	Putative: Distal small intestine and colon	MASLD (Madsen et al., 1976; Kuang et al., 2023)
SCFAs			
Acetate	*Akkermansia muciniphila*, *Bacteroides spp.*, *Bifidobacterium spp.*	Colon	IBD, CRC (Imhann et al., 2018; Louis et al., 2014; Macfarlane and Macfarlane, 2003)
Propionate	*Coprococcus catus*, *Eubacterium hallii*, *Bacteroides spp.*	Colon	IBD, CRC (Louis et al., 2014; Macfarlane and Macfarlane, 2003; Sun et al., 2017)
Butyrate	*Coprococcus comes*, *Coprococcus eutactus*, *Anaerostipes spp.*	Colon	IBD, CRC (Louis et al., 2014; Macfarlane and Macfarlane, 2003; Sun et al., 2017)
Tryptophan metabolites			
Indole	*Achromobacter liquefaciens*, *Bacteroides ovatus*, *Bacteroides thetaiotamicron*	Putative: colon	IBD, metabolic syndrome, obesity (Agus et al., 2018; Devlin et al., 2016)
Indole derivatives	*Bacteroides spp.*, *Clostridium spp.*, *Escherichia coli*	Putative: colon	Metabolic syndrome, obesity (Agus et al., 2018; Devlin et al., 2016)
Kynurenines’	*Lactobacillus spp., Pseudomonas aeruginosa, Pseudomonas fluorescens*	Putative: colon	IBD, IBS, metabolic syndrome, obesity (Agus et al., 2018; Vujkovic-Cvijin et al., 2013)
Tryptamine	*Clostridium sporogenes*, *Ruminococcus gnavus*	Putative: colon	Depression, ASD (Kałużna-Czaplińska et al., 2019; Williams et al., 2014)
Serotonin (5-hydroxytryptamine)	*Indigenous spore-forming bacteria*	Putative: colon	IBS, metabolic syndrome, obesity, depression, ASD (Fung et al., 2019; Kałużna-Czaplińska et al., 2019; Yano et al., 2015)
Imidazole propionate	*Aerococcus urinae*, *Streptococcus mutans*, *Anaerococcus prevotii*, *Adlercreutziae equolifaciens*, *Eggerthella lenta*, *Lactobacillus paraplantarum*, *Brevibacillus laterosporus*, *Shewanella oneidensis*	Putative: colon	T2DM (Koh et al., 2018)
Others			
Dopamine	*Enterococcus*, *Lactobacillus*	Jejunum	Parkinson’s disease (van Kessel et al., 2019)
Dopamine	*Enterococcus faecalis*, *Enterococcus faecium*	Intestine	Parkinson’s disease (Maini Rekdal et al., 2019)
Histamine	*Morganella morganii*, *Escherichia coli*	Intestine	Asthma (Barcik et al., 2017)
TMAO	*Clostridia*, *Proteus*, *Shigella*, and *Aerobacter*	Colon	CVD (Gatarek and Kaluzna-Czaplinska, 2021; Subramaniam and Fletcher, 2018)

DCA, deoxycholic acid; LCA, lithocholic acid; UDCA, ursodeoxycholic acid; HDCA, hyodeoxycholic acid; CRC, colorectal cancer; HCC, hepatocellular carcinoma; PBC, primary biliary cirrhosis; PSC, primary sclerosing cholangitis; MASLD, metabolic-associated steatotic liver disease; IBD, inflammatory bowel disease; IBS, irritable bowel syndrome; ASD, autism spectrum disorder; TMAO, trimethylamine N‐oxide; CVD, cardiovascular disease.

### Stomach

About forty years ago, the stomach was generally regarded as a microbiologically sterile organ because its microenvironment was considered inhospitable for bacterial growth (Yang et al., 2013). With the advancement of modern technology, it was gradually recognized that some specific bacteria existed in the stomach (i.e., *Helicobacter pylori*), and the abnormal increase of certain pathogenic bacteria can lead to the occurrence of diseases (de Vos et al., 2022; Hunt et al., 2015). The microenvironment of acidity pH, high levels of digestive enzymes, oxygen content, and peristalsis speed resulted in relatively lower bacterial abundance (Hunt et al., 2015). Therefore, the α diversity was decreased and the aero- and acid-tolerant taxa were enriched in the healthy stomach. Dominant members of the phylum bacteria in the stomach were Proteobacteria, Firmicutes, Bacteroidetes, Fusobacteria, and Actinobacteria (Kaźmierczak-Siedlecka et al., 2022). The gut microbiome composition and functions varied along the gastrointestinal tract. Besides, studies have suggested that the crosstalk between the gut microbiome of the stomach and the microbiota in the lower gastrointestinal tract contributes to disease development. The patients carried *Helicobacter pylori* also had an extremely low relative abundance of several Bifidobacterium species in the lower gut of those with aggressive gastric diseases (Devi et al., 2021). Bile reflux gastritis and the higher intragastric pH values have been implicated with the etiopathogenesis of gastritis and gastric cancer, which lead to the elevated concentration of conjugated and secondary BAs (Li et al., 2019; Wang et al., 2022) and the enrichment of pathogenic bacterium (Jin et al., 2022a; Noto et al., 2022). Besides, several oral pathogenic bacteria were related to the persistence of atrophy and intestinal metaplasia in the stomach, such as *Peptostreptococcus*, *Streptococcus*, *Parvimonas*, *Prevotella*, *Rothia*, and *Granulicatella* (Kaźmierczak-Siedlecka et al., 2022, Rajilic-Stojanovic et al., 2020). The occurrence of pathogenic bacterial thriving in patients with bile reflux and the translocation of oral pathogens both contributed to the etiopathogenesis of gastric or other gastrointestinal disorders, which also provides us with an idea to treat the related diseases by focusing on bacterial longitudinal displacement.

### Duodenum

The duodenum received the chyme from the stomach and alternately contracted, and relaxed, which moved the food to the following intestinal segments. The contraction of the duodenum helped the product in the intestine transported to the jejunum quickly. Primary bile acids were synthesized from the cholesterol in the liver and temporarily stored in the gallbladder (Jia et al., 2018). After the meal, they were secreted into the duodenum with pancreatic juices to facilitate digestion and absorption of dietary lipids and nutrients (Jia et al., 2018). Meanwhile, bile acids had certain bactericidal effects, as the main regulator of microbial activity (An et al., 2022). Besides, due to the relatively higher oxygen content in this intestinal segment, coupled with the presence of gastric acid and various digestive juices, and the shorter transit time, the abundance of the duodenal bacterial load is relatively low, approximately between 10^2^ and 10^4^ CFU/mL (Simon and Gorbach, 1984). Limited by sampling methods, the study focused on the small intestinal flora, especially the duodenal flora was much more difficult than that of feces. As for the microbial composition, the common duodenal microbiota phyla include Firmicutes, Proteobacteria, and Actinobacteria (Sroka-Oleksiak et al., 2020). Shanahan et al. confirmed the presence of duodenal mucosa-associated microbiota, dominated by the genus of *Streptococcus*, *Prevotella*, *Veillonella*, and *Neisseria* in the lower level, with newly developed encased biopsy forceps (Shanahan et al., 2016).

Studies have shown that elevated pH levels resulting from hypoacidity and reduced BA secretion can lead to bacterial overgrowth in the duodenum, including many oral pathogenic bacteria, such as *Rothia mucilaginosa*, *Streptococcus salivarius* and *Granulicatella adiacens* (Filardo et al., 2022). This suggested a disordered longitudinal distribution of the oral microbiota to the distal gut, resulting in duodenal dysbiosis and pathological conditions. Another study reported that the oral normal microflora like *Shuttleworthia* and *Rothia* are overrepresented at the distal duodenum in patients with alcoholic liver disease, implying that some microbes shifting down into the duodenum was implicated in the progression of alcoholic liver disease (Maccioni et al., 2020). Besides, the gut microbial structure of duodenal mucosa was closely related to type 2 diabetes (T2DM) and obesity (Darra et al., 2023; Sroka-Oleksiak et al., 2020). Emmanouil et al. compared the duodenal microbiota samples of the patients with obesity and healthy controls through 16S rDNA sequencing and found that obese individuals had a significant increase in anaerobic genera (Angelakis et al., 2015). Meanwhile, the metabolism pathway of glycerophospholipid and the pathway of sucrose phosphorylase were downregulated in obese individuals (Angelakis et al., 2015). Furthermore, bariatric surgery and duodenal mucosal resurfacing (DMR) surgery showed that the proximal small intestine is the key contributor to postoperative metabolic benefits, which involved the displacement of duodenal microbiota (Liou et al., 2013; van Baar et al., 2021). It is worth noting that, diets induced mucosa-associated microbiota dysbiosis varied across the intestinal tract. Studies based on the porcine metabolic syndrome model have shown that *Lactobacillus johnsonii*, which had an anti-obesity effect by preventing inflammation and mucosal barrier disruption (Yang et al., 2020), was elevated at the duodenum and decreased at the cecal and rectal luminal regions (Xu et al., 2022).

### Jejunum

The intestinal segment of the jejunum is an important site for the digestion and absorption of dietary lipids. Dietary lipids digested in the duodenum are further absorbed in the jejunum as fatty acids. The small intestine especially the jejunum was responsible for the majority of dietary nutrients digestion and absorption, also having profound effects on the host physiology (El Aidy et al., 2015; Ermund et al., 2013). However, few studies have investigated the composition of intestinal flora in the jejunum because of the limited access to obtain jejunal intestinal contents or tissue in healthy individuals, and most of the investigations have focused on the large intestine or feces. Complex interactions of oxygen level, nutrient bioavailability, pH, bile acid contents, gastrointestinal motility, mucus, and immune factors contribute to the low levels of diversity and richness of gut bacteria in the jejunum, approximately between 10^4^ and 10^7^ CFU/mL (Simon and Gorbach, 1984). The common sampling method such as the endoscope needed to pass through the oral cavity and upper gastrointestinal tract, which will cause confusion and contamination of samples. In a study investigating the jejunal bacterial microbiota from fasting obese patients by surgery, the jejunal bacteria richness was relatively lower, approximately 10^3^–10^4^ CFU/mL (Villmones et al., 2022). Under normal physiological conditions, the intestinal barrier of the jejunum protects the internal circulation environment from destruction by pathogenic intestinal bacteria, including physical barriers (epithelial cells, tight junctions), chemical barriers (mucus layer, antimicrobial peptide), immunological barriers (such as secretory immunoglobulin A (sIgA)), and the competitive effect of commensal microbiota. Cani et al. observed an increase in circulating lipopolysaccharides (LPS) level in mice fed a high-fat diet (HFD) for 4 weeks, which further contributed to the increased inflammatory response during obesity development. This metabolic alteration occurs because of the disruption of the jejuno-intestinal physical barrier (Cani et al., 2007).

### Ileum

The ileal microbial load was estimated 10^7^–10^8^ CFU/mL compared with the upper intestinal tract, accompanied by increased facultative and strict anaerobes (Hayashi et al., 2005). As for the ileal-harbored bacteria, most taxa of the Enterobacteriaceae and the Bacilli class of the Firmicutes, and relatively high abundances of Enterococcus, Lactobacillus, Clostridium, Streptococcus were found in ileum from human samples (Ahmed et al., 2007; Booijink et al., 2010; Donaldson et al., 2016; Hayashi et al., 2005; Wang et al., 2005). Similarly, the compositional and functional fluctuations of the lower small intestinal microbes were largely unexplored due to the inaccessibility of the biological specimens. Recently, researchers found that the human gut microbiota in the distal small intestine would react flexibly like changing the biomass of the whole microbes or the proportions of subspecies in response to the nutritional status, providing a novel understanding of the host-gut microbiome crosstalks (Yilmaz et al., 2022).

Beyond the roles of digestion and absorption of dietary nutrients, the ileum was widely involved in regulating the host’s metabolic homeostasis through the gut microbiome-host interactions. BAs, synthesized by the hepatic enzymes from cholesterol, were the key participants. The hepatic primary BAs would conjugate taurine or glycine, forming the conjugated primary BAs, then were secreted into the intestinal tract when the gallbladder contracted after meals. The conjugated BAs would be deconjugated by the bile salt hydrolase (BSH) from the gut microbiota in the ileum. And the bile acids that reached the lower small intestine would be reabsorbed into the portal vein by passive and active transport including the apical sodium bile acid transporter (ASBT) and recirculated to the liver (Jia et al., 2018).

Many nuclear receptors were expressed in the distal ileum, including farnesoid X receptor (FXR), G-protein-coupled bile acid receptor 1 (GPBAR1, TGR5), G-protein-coupled receptor 43 (GPR43), and vitamin D receptor (VDR)(Fiorucci et al., 2021). The intestinal FXR-liver axis played an important role in the regulation of hepatic bile acid synthesis and glycolipid metabolism (Sun et al., 2021). The fibroblast growth factor 15/19 (FGF15/19) was released from the ileal enterocytes when the FXR signaling was inhibited, further, the hepatic cholesterol 7α-hydroxylase (CYP7A1), the rate-limiting enzyme of the bile acid synthetic pathway, was downregulated (Inagaki et al., 2005). Besides, the bile acid-intestinal FXR-ceramide axis also played a part in glucose and lipid metabolism, preventing the development of metabolic-associated steatotic liver disease (MASLD), insulin resistance, and even diabetes when the intestinal FXR signaling was depleted (Chen et al., 2022; Gonzalez et al., 2016; Wu et al., 2021). TGR5 was another BA-binded receptor activated by multiple conjugated and unconjugated BAs, the glucagon-like-peptide 1 (GLP-1) would be released when the TGR5 in enteroendocrine L cells was activated (Ahmad and Haeusler, 2019; Pols et al., 2011). Hyocholic acid species (HCAs), a special group of BAs from pig, could strongly stimulate the secretion of GLP-1 through a distinct inhibited FXR and activated TGR5 mechanism in enteroendocrine cells simultaneously (Makki et al., 2023; Zheng et al., 2021) to promote insulin secretion and further maintain glucose homeostasis (Albaugh et al., 2019), besides, ileal FXR signaling pathway was involved in the therapeutic effects of hyodeoxycholic acid (HDCA) for MASLD (Kuang et al., 2023). Another endogenous bile acid, cholic acid-7-sulfate (CA7S), increased after the sleeve gastrectomy, inducing the GLP-1 secretion by activating the TGR5 (Chaudhari et al., 2021). Besides, short-chain fatty acids (SCFAs) also could trigger the secretion of GLP-1 from intestinal L cells by activating the GPR43/41, exerting beneficial effects on obesity and diabetes (Tolhurst et al., 2012). L-arabinose, a microbiota-accessible carbohydrate (MAC), could increase the relative abundance of microbiota-derived SCFAs, which will further activate the GPR43/GPR41 from the distal ileum (Tomioka et al., 2022). Another nuclear receptor, VDR, was highly expressed in the ileum, and activated by the lithocholic acid (LCA) and 3-oxo-LCA, 6-oxo-LCA (Makishima et al., 2002). LCA would increase the ileal expression of *Cyp24a1* and *Cyp3a11*, which were responsible for the detoxification of LCA (Adachi et al., 2005; Ishizawa et al., 2018). Besides, lack of the VDR in Paneth cells would contribute to the impaired anti-bacterial capability, increasing the inflammatory responses (Lu et al., 2021).

### Colon

The thicker colonic outer and inner mucus layers allowed the inhabitant of larger biomass of anaerobic microbiota up to 10^12^–10^14^ CFU/mL compared with the small intestine (Sommer and Bäckhed, 2013). Given the microenvironment and physiological properties, the colon was enriched in the families *Bacteroidaceae*, *Prevotellaceae*, *Lachnospiraceae*, and *Ruminococcaceae* (Flint et al., 2012).

In a physiological state, the majority of dietary carbohydrates, lipids, and proteins would be emulsified, digested, and absorbed in the small intestine, and few would escape this process reaching the colon. Besides, the un-digestible dietary fibers would pass through the gastrointestinal tract all the way to the colon because of the lack of host enzymes that could break down the fibers. Under the fermentation of dietary carbohydrates by some bacteria including genera *Bacteroides*, *Prevotella*, *Parabacteroides*, and *Alistipes* (Flint et al., 2015), the microbiota-derived SCFA could fuel the colonocytes, mitigate the inflammation, and served as signaling molecules (Koh et al., 2016). The deficiency of SCFA was closely related to the progression of diabetes, and supplementation of SCFA producers could alleviate the disturbance of glucose metabolism to some extent (Zhao et al., 2018), which implied targeting the dietary fibers-gut microbiota-SCFA to treat metabolic diseases would be a feasible approach (Canfora et al., 2019).

The colon was the dominant site for the secondary metabolism of the primary BAs that escaped from the enterohepatic circulation in the distal ileum. After the deconjugation of conjugated BAs by BSH, primary unconjugated BAs like cholic acid (CA) and chenodeoxycholic acid (CDCA) would be 7α-dehydroxylated to form the deoxycholic acid (DCA) and LCA respectively by a series of BA-inducible (bai) enzymes from *C. scindens*, *C. hiranonis* and *C. hyelomonae* (Funabashi et al., 2020; Ridlon et al., 2016a). Another commensal bacteria *Faecalicatena contorta S122* was recently found to efficiently convert CA/CDCA to DCA/LCA respectively (Jin et al., 2022b). Apart from the deconjugation, and dehydroxylation forms of BA transformation, oxidation and epimerization were also the other secondary metabolisms of BAs by gut microbiota (Devlin and Fischbach, 2015). A large number of oxo-, epi- and iso-BA derivatives metabolized by gut microbiota have been identified, recent studies reported that LCA could be metabolized into the trace cluster of BA, 3-oxoLCA, and isoLCA by the gut microbiota isolated from human feces, including 12 bacterial genera (Song et al., 2020). Another study identified a special secondary BA, isoalloLCA, produced by the *Odoribacteraceae* strains after which were screened in the centenarians (Sato et al., 2021). As for BA epimerization, the microbial 7α-hydroxysteroid dehydrogenase (7α-HSDH) and 7β-HSDH could convert the CDCA to ursodeoxycholic acid (UDCA) (Heinken et al., 2019) and DCA to iso-DCA (Song et al., 2021) respectively.

## GI locations: determinant of microbial properties in health and diseases

In recent decades, microbiome research has primarily focused on the large intestine (colon) and fecal samples, revealing their role in maintaining metabolic homeostasis. However, emerging evidence suggests that the upper intestinal regions, particularly the small intestine (duodenum, jejunum, and ileum), plays a significant role in this balance. Studies have found distinct microbiota in the jejunum and ileum compared to the colon (Zmora et al., 2018), with the proximal small intestine (stomach and duodenum) showing greater response to external stimuli than the distal small intestine (jejunum and ileum) (Seekatz et al., 2019). Additionally, research has shown rapid changes in ileal microbiota biomass and sub-strains in response to feeding status compared to the relatively stable colonic microbiota (Yilmaz et al., 2022). These findings warrant in-depth investigation into the role of the small intestine in regulating host metabolism.

The distribution of the microbiota is closely related to its function and can significantly impact host metabolism in both normal and abnormal conditions (Tropini et al., 2017). The circadian rhythm, a fundamental physiologic process, is regulated by core clock transcription factors, which in turn affects cellular processes and helps the body adapt to daily oscillations (Roenneberg and Merrow, 2016; Takahashi, 2017). Disturbances in the diurnal rhythmicity of host-gut microbiota interactions contribute to metabolic dysfunctions (Thaiss et al., 2015, 2016). Studies have shown that a HFD can cause changes in the composition and gene expression of the microbiota in the small intestine, particularly the ileum, which plays a crucial role in regulating host circadian rhythms (Dantas Machado et al., 2022). HFD also impairs the expression of the antimicrobial peptide Reg3γ in the distal small intestine, disrupting the rhythmicity of the gut microbiota and leading to metabolic dysregulation (Frazier et al., 2022). Additionally, recent research has demonstrated that diet can modulate the function of the small intestine barrier through the diet-microbiota-small intestine MHC class II-IL-10 axis, highlighting its importance in developing interventions for gut inflammatory diseases (Tuganbaev et al., 2020). Interestingly, targeting microbial choline trimethylamine lyase inhibition has been shown to improve metabolic status by reshaping circadian rhythms (Schugar et al., 2022).

In various pathological conditions, the gut microbiota has been closely linked to a cluster of diseases, such as obesity, diabetes, MASLD, inflammatory bowel disease (IBD), irritable bowel syndrome (IBS), and celiac disease (CeD) (de Vos et al., 2022).

In IBD, chronic inflammation affects the entire GI tract and is accompanied by disruptions in gut microbiota composition. A recent review has highlighted the importance of reclassification of Crohn’s disease (CD) into ileum-dominant and colon-dominant types based on site-specific changes in pathophysiological characteristics (Atreya and Siegmund, 2021). However, the contribution of the small intestine to IBD pathogenesis, particularly in CD, has been overlooked. An emerging study revealed that the microbial composition of the colon partially resembles that of the small intestine, including specific pathobionts found in the small intestine that are relevant to disease phenotypes (Ruigrok et al., 2021). This suggests that metabolic dysregulation in the small intestine may precede and potentially contribute to colonic disorders.

Small intestinal bacterial overgrowth (SIBO) occurs when there is an overload of non-native or native bacteria in the duodenum and jejunum. SIBO is closely associated with conditions such as IBS, MASLD, and T2DM, characterized by abnormal concentrations of bacteria in the upper small intestine and corresponding clinical manifestations (Skrzydło-Radomańska and Cukrowska, 2022). Many factors contributing to the occurrence of SIBO, including gastric acid secretion deficiency, bilio-pancreatic insufficiency, anatomical modifications by surgery, intestinal dysmotility, impaired intestinal mucosal integrity, and a dysfunctional ileocecal valve, have been systematically reviewed elsewhere (Bushyhead and Quigley, 2022). SIBO has a relatively high prevalence in gastrointestinal disorders like IBS, IBD, and CeD. In metabolic diseases, obesity is linked to an elevated risk of SIBO, with a prevalence rate of 26% in obese individuals compared to 6% in healthy controls (Ierardi et al., 2016; Roland et al., 2018). SIBO may play a contributing role in the onset and development of obesity. Additionally, approximately 35% of individuals with chronic MASLD exhibit SIBO, indicating its presence throughout different stages of liver diseases (Gudan et al., 2022). Increased intestinal permeability is associated with a higher incidence of SIBO in MASLD patients, suggesting a potential role in the pathogenesis of MASLD (Miele et al., 2009). The mechanisms by which SIBO contributes to MASLD need further investigation. Pathophysiological influences of SIBO include mucosal injury induced by bacteria and competition for dietary nutrients, leading to maldigestion and malabsorption (Bushyhead and Quigley, 2022). Recent research has shown that individuals with SIBO have reduced microbial diversity and disruptions in the ecological network of the duodenal microbiota, with notable elevations of *E. coli* and *Klebsiella* strains, potentially triggering inflammatory immune response (Leite et al., 2023). Therefore, therapeutic strategies for metabolic diseases with concurrent SIBO should focus on reconstructing the small intestinal microbiota to restore the regulation of metabolites and the intestinal immune-mediated metabolic signaling pathways.

It is important to note that the relationship between SIBO and disease remains a subject of ongoing debate, raising the question of whether SIBO contributes to the onset of certain diseases or as a consequence of underlying pathologies. This “chicken or egg” situation complicates our understanding of SIBO’s role in disease progression. Conditions such as impaired gut motility, immune dysfunction, or altered intestinal anatomy may predispose individuals to SIBO, suggesting that it could be a secondary effect. However, SIBO itself may exacerbate disease by promoting inflammation or disrupting normal nutrient absorption. Further research, particularly longitudinal studies, is needed to clarify this bidirectional relationship and determine whether SIBO is a driver of disease or a consequence.

CeD, an autoimmune enteropathy mediated by T cells, occurs in genetically predisposed individuals with the absence of gluten metabolism-related genes (Abadie et al., 2011). A longitudinal prospective cohort study examining fecal samples from children at high risk of developing CeD revealed that altered microbial signatures occurred before the onset of the disease (Leonard et al., 2021). Notably, the predominant site of CeD is localized in the small intestine, where specific compositional and functional changes in the duodenal microbiota have been linked to impaired gluten degradation (Caminero et al., 2016; Constante et al., 2022). These findings emphasize that the GI location is a determinant for the composition and functionality of microbiota in CeD.

**Figure 2.**
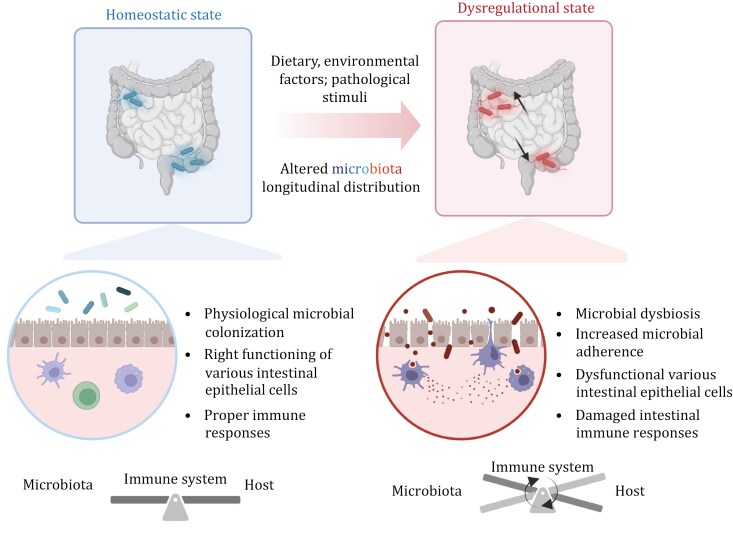
The disruption of gut longitudinal microbial distribution contributed to the host metabolic dysregulation. Various dietary, environmental, and pathological stimuli would lead to the altered GI longitudinal microbiota distribution, which disrupts the microbiota–metabolites–immune response homeostatic state.

The pivotal role of GI location and its resident microbiota in the development and progression of diseases, such as MASLD, T2DM, obesity, and other GI disorders, needs to be reevaluated. The spatial distribution of gut microbiota along the GI tract has profound effects on host physiology by influencing the microbiota–metabolites–immune system interaction (Fig. 2). Understanding the longitudinal shifts of gut microbiota along the GI tract during disease progression is essential for conducting mechanistic studies and developing effective intervention approaches.

## Disrupted microbial distribution in metabolic disease onset: insights from bile acid metabolism

BAs are synthesized from cholesterol in the liver through a series of complex enzymatic pathways. After a meal, BAs are secreted into the small intestine along with pancreatic enzymes and undergo further metabolism by the gut microbiota, leading to the diversification of the BA pool. In the physiological state, microbial transformations of BAs, such as deconjugation, hydroxylation, and epimerization. These transformations play a crucial role in nutrient absorption and maintaining intestinal metabolic and immune homeostasis.

**Figure 3.**
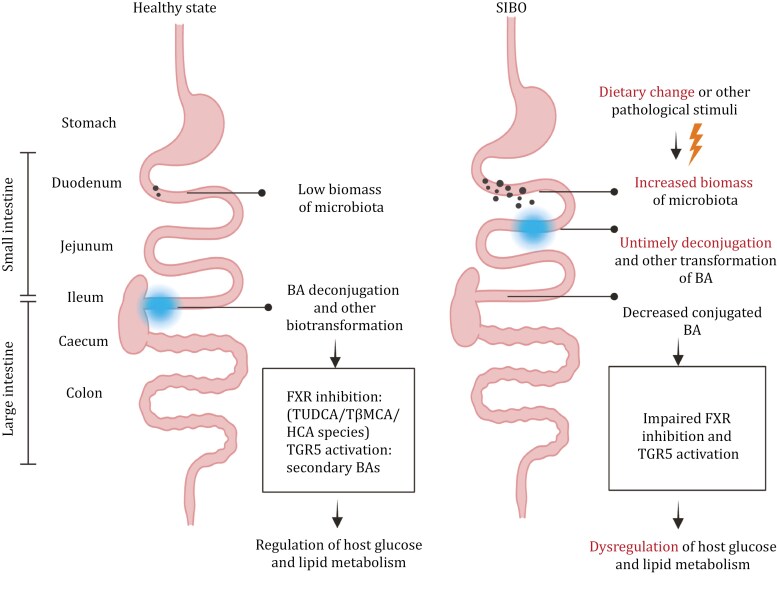
Dislocation of microbiota contributes to bile acid-mediated impaired host metabolism: a scenario that possibly happens in SIBO. Bile acids are normally metabolized (deconjugation and other biotransformation) in the lower small intestine, especially in the terminal ileum. When the SIBO occurs, bile acids are untimely deconjugated and transformed by the overgrown microbiota in the upper small intestine, which results in the less conjugated bile acids reaching the terminal ileum, impairing the regulation of bile acids on the glucose and lipid metabolism by targeting the critical receptors such as FXR and TGR5. BA, bile acid; FXR, farnesoid X receptor; TGR5, G-protein-coupled bile acid receptor 1; TUDCA, tauroursodeoxycholic acid; TβMCA, tauro-β-muricholic acid; HCA, hyocholic acid.

Changes in dietary patterns or other external pathophysiological factors can significantly alter the BA profile, affecting both its biosynthesis and secretion processes (Zheng et al., 2017). These alterations can reduce the anti-bacterial effect of BAs in the upper small intestine, allowing certain microbial populations to thrive in these regions and deviate from their original ecological niches, potentially contributing to SIBO (Fig. 3). The displacement of microbiota can cause the deconjugation of conjugated BAs in advance, resulting in a reduced proportion of conjugated BAs reaching the distal small intestine, while untimely deconjugated BAs may diffuse into the circulation via passive transport. These changes in the BA profile disrupt the regulatory effects of conjugated/unconjugated and primary/secondary BAs on BA-related receptors, leading to the malfunction of BA-mediated host glucose and lipid metabolism by disrupting intestinal FXR signaling and the TGR5-GLP-1 axis. Additionally, the displacement of microbiota can trigger intestinal immune responses, disrupting the homeostasis of the intestinal immune system and inducing intestinal inflammation in the proximal small intestine. These dysregulations may further fuel the progression of various metabolic diseases, including obesity, T2DM, MASLD, and GI disorders.

Recently, studies have identified novel microbial-mediated biosynthetic conjugated forms of BAs (Mohanty et al., 2024a; Ridlon and Gaskins, 2024) in the intestine, such as acylated- (Liu et al., 2024; Nie et al., 2024),amino acid conjugated- (Quinn et al., 2020; Rimal et al., 2024) and polyamine conjugated BAs (Mohanty et al., 2024b), with unexpectedly high levels observed in the small intestine, although their precise physiological functions still require further elucidation. Importantly, whether dislocated microbiota in the proximal small intestine could lead to the production of these newly identified and yet more uncharted forms of BAs remain unclear. Additionally, the existence of regional-specific microbiota-mediated BA metabolism along the GI tract warrants further exploration and detailed investigation.

## Targeting the redistribution of gut microbiota for preventive and therapeutic intervention

**Figure 4.**
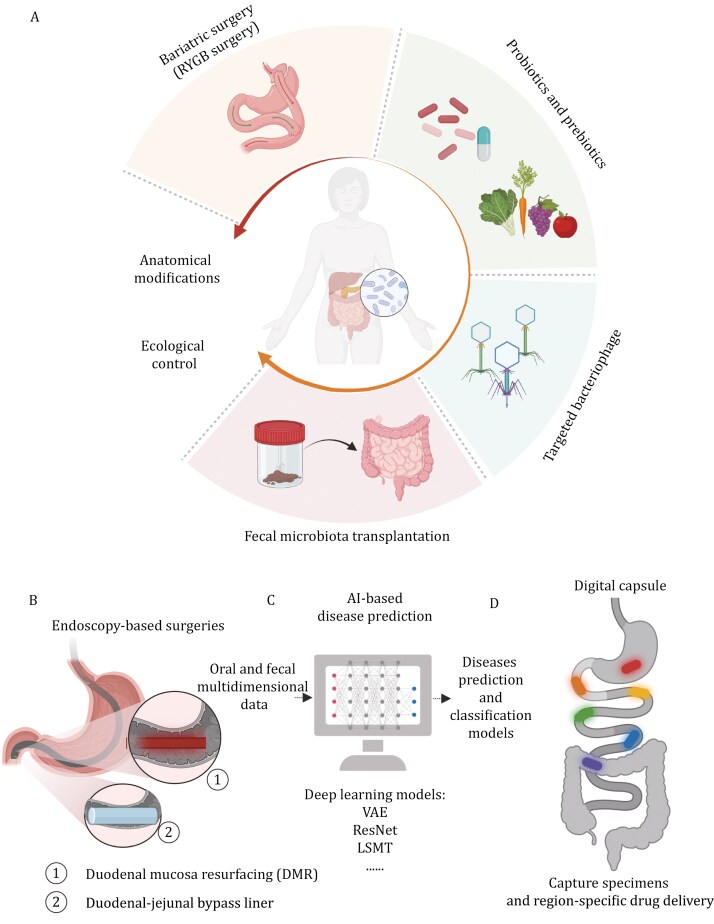
Ways to redistribute the gut longitudinal microbiota. (A) The traditional ways to redistribute the GI longitudinal microbial distribution including RYGB surgery, probiotics/prebiotic intervention, targeted bacteriophage, and FMT. (B) Endoscopy-based surgeries including duodenal mucosa resurfacing (DMR) (hydrothermal ablation of duodenal mucosa) and duodenal–jejunal bypass liner (endoscopically implanted 60 cm impermeable fluoropolymer sleeve) procedures to functionally deprive the duodenal mucosa. (C) Employing the oral and fecal microbial multidimensional data to construct artificial intelligence (AI)-based deep-learning prediction models. (D) Digital capsules to capture regional-specific human GI biospecimens and deliver drugs.

This review outlines different strategies for modulating the distribution of gut microbiota along the GI tract, providing insights and guidance for the development of effective therapeutic approaches that target the longitudinal displacement of gut microbiota. These therapeutic strategies can be broadly classified into two main categories: anatomical modifications of the microbiota through surgery and ecological control achieved by introducing exogenous bacteria or metabolites into the gut ecosystem (Fig. 4A). Moving forward, we aim to explore innovative interventional and therapeutic strategies that target the manipulation of longitudinal gut microbial distribution, which merit further investigation and attention.

### Anatomical modifications

How does gastric bypass surgery specifically alter the microbiota composition of the small intestine? Bariatric surgery has emerged as a highly effective treatment strategy for obesity and T2DM, leading to significant reductions in body weight, blood glucose levels, and improved insulin sensitivity (Kirwan et al., 2022; Mingrone et al., 2021; Schauer et al., 2017). The two most commonly performed bariatric surgeries are vertical sleeve gastrectomy (VSG) and Roux-en-Y gastric bypass (RYGB). VSG involves the removal of a large portion of the stomach, while RYGB re-routes the intestinal tract, bypassing the stomach, duodenum, and proximal jejunum. Notably, the postoperative metabolic improvements have been linked to changes in BAs profiles and gut microbiota composition in both human and rodent models. These metabolic changes include elevated levels of total and specific serum BAs (McGavigan et al., 2017; Myronovych et al., 2020; Patti et al., 2009; Ryan et al., 2014), as well as alterations in the composition and function of the gut microbiota, which may contribute to improved glucose metabolism (Dang et al., 2021; Fouladi et al., 2019). A recent study demonstrated that germ-free mice receiving gut microbiota from post-RYGB surgery patients on a Western diet exhibited enhanced insulin sensitivity, characterized by a reshaped gut microbiota structure (increased *Akkermansia muciniphila* or *Blautia*) and metabolomic profiles (increased tryptophan-derived metabolites) (Yadav et al., 2023). The gut microbiota appears to be a key determinant in the regulation of glucose metabolism following RYGB (Debédat et al., 2022). More importantly, we reckoned that bariatric surgery especially RYGB, specifically and significantly altered the microbiota composition of the small intestine owing to the more rapid and pronounced changes in nutrient flow, bile acid metabolism, gut transit time, and oxygen levels compared with distal intestine. The anatomical alteration reduced exposure of ingested food to gastric acids, digestive enzymes, and BAs in the bypassed section, creating an environment less favorable for certain bacteria while promoting others. The reduction in bile acid exposure in the upper small intestine can decrease the growth of bile-sensitive bacteria and increase bile-resistant ones. Besides, RYGB accelerated the transit of food through the small intestine, reducing the time food spent in this region. This limited the growth of bacteria that rely on slow transit and fermentation. These alterations led to shifts in bacterial populations, favoring species that adapt to the new metabolic and environmental conditions, ultimately contributing to improved metabolic health and weight loss.

### Ecological control

From an ecological perspective, the introduction of exogenous agents such as probiotics, prebiotics, phages, and fecal microbiota transplantation (FMT) can compete with the dislocated microbiota for nutrition, thereby regulating the ecological balance and facilitating the decolonization of pathogenic or dislocated microbiota from their ecological niches. This process not only promotes the re-establishment of beneficial microbial communities but also has the potential to alleviate metabolic diseases and GI disorders.

FMT has emerged as a promising therapeutic approach for GI diseases, particularly for recurrent *Clostridioides difficile* infection (rCDI), where it has demonstrated a high therapeutic response (Hui et al., 2019; Ianiro et al., 2018; Quraishi et al., 2017). The efficacy of FMT is not only dependent on the microbial composition and functions of the donor microbiota in the recipient (Porcari et al., 2023), but also on the specific GI colonization site, which significantly impacts therapeutic outcomes. For instance, FMT using wild boar microbiota has been shown to modulate the structure of jejunal gut microbiota and alleviate HFD-induced obesity (Zhu et al., 2022). Additionally, self-reinoculation in rodents significantly altered the composition and function of the gut microbiota in the small intestine, with less pronounced effects on the large intestine, while unconjugated BAs were found to be predominant in the small intestine of non-coprophagic mice (Bogatyrev et al., 2020). In another study, whole-intestinal microbiota transplantation (WIMT) from pigs was found to better promote small intestinal epithelial development and reduce systemic inflammatory response compared to conventional FMT, which predominantly represents gut microbiota from the large intestine (Li et al., 2020). Therefore, these findings underscored the fact that applying fecal microbiota to reconstitute the intestinal microbial ecology in patients is inappropriate, given the significant differences in microbiota distribution along the GI tract. It also highlighted the importance of the specific GI colonization site in determining the efficacy of FMT. Additionally, this suggests that WIMT may be a more effective approach in the future for reconstructing a healthy ecological relationship between host and microbiota.

The efficacy and heterogeneity of probiotics, prebiotics, and phage-based therapies across different regions of the GI tract have been extensively reviewed (Sorbara and Pamer, 2022; Strathdee et al., 2023). To enhance their effectiveness and minimize potential adverse effects, it is crucial to develop targeted microbiota or drug delivery systems that enable site-specific release and action. The design and modification of bioinspired oral delivery systems, which can improve efficacy, have been comprehensively reviewed elsewhere (Zhang et al., 2023). A cutting-edge study recently employed an advanced 18-strain commensal bacterial consortium, named the “F18-mix,” to target and eliminate pathogenic Enterobacteriaceae strains, specifically *Klebsiella pneumoniae* and *Escherichia coli*, in the gut (Furuichi et al., 2024). These pathogenic bacteria often persist in the intestinal tract due to disruptions caused by antibiotic treatments, leading to inflammatory conditions such as IBD. The application of the F18-mix represents a promising alternative to traditional antibiotic therapies, particularly in combating infections caused by drug-resistant strains of *Klebsiella* and *E. coli*. Furthermore, the development of personalized probiotic interventions customized to an individual’s unique gut microbial composition and dietary patterns holds great potential for optimizing gut health and proactively preventing pathogen overgrowth in the future.

## Future direction

Recent advancements in endoscopy-based interventional approaches provide a novel therapeutic strategy for T2DM, which has garnered significant attention. The duodenum is increasingly recognized as the metabolic center, and Western diets high in fat can cause hyperplasia of the duodenal mucosa, leading to impaired lipid and glucose metabolism (Ghosh et al., 2018; Gniuli et al., 2010; Nguyen et al., 2015; Rubino et al., 2006). The DMR procedure, which aims to ablate the duodenal mucosa through hydrothermal means, has emerged as a potential new treatment for glycemic control. Multiple international multi-center DMR clinical trials have demonstrated the efficacy of DMR surgery in durably improving insulin sensitivity and multiple metabolic indicators such as HbA1c and HOMA-IR in T2DM patients, without reported adverse effects to date (Mingrone et al., 2022; van Baar et al., 2020, 2022). Notably, changes in the longitudinal distribution of gut microbiota may contribute to the morphological and functional reshaping of the duodenal mucosa, although the causal relationship between them remains elusive and requires further investigation. Additionally, glycemic remission after DMR surgery may potentially be linked to the suppression of microbial overgrowth and the rectification of its subsequent detrimental effects on mucosal immune and glycolipid metabolism. Another technique duodenal–jejunal bypass liner shows promising clinical outcomes in treating T2DM and obesity by mimicking the functional deprivation of proximal small intestine of RYGB surgery while being less invasive (Ryder et al., 2023). Studies have demonstrated significant reductions in body weight, HbA1c levels, systolic blood pressure, and cholesterol in patients who have undergone this treatment (Ryder et al., 2023). Additionally, it has led to reduced insulin dosages or even discontinuation for some patients. Despite these positive results, there are safety concerns, particularly related to gastrointestinal bleeding and liver abscess formation, which have led to early removal in some cases. Nevertheless, with proper monitoring, the risks appear manageable. Looking forward, duodenal–jejunal bypass liner has the potential to become a widely available treatment option for patients with refractory uncontrolled T2DM and obesity, especially as demand for noninvasive metabolic treatments grows. Consequently, the restoration of regional-specific host-microbiota homeostasis through endoscopy-based therapeutic approaches holds great promise in the treatment of metabolic disorders (Fig. 4B).

The limited accessibility of human biospecimens from the upper GI tract, such as the duodenum and jejunum, necessitates more feasible methods for early detection and intervention in cases of disrupted gut microbial distributions. Recent research has shown the similarities between oral and fecal microbiota, and has shown that changes in oral microbial composition often precede the clinical manifestations of diseases (Yang et al., 2023). These findings suggest that oral microbes may serve as potential biomarkers for early-stage disease detection. Integrating multidimensional data from oral and fecal samples into disease prediction models can help depict microbial trajectories during disease progression (Hernández Medina et al., 2022). Techniques like variational autoencoders (VAE) (Allesøe et al., 2023; Gomari et al., 2022; Nissen et al., 2021), and residual neural networks (ResNet) are utilized for this purpose (Buergel et al., 2022; Michel-Mata et al., 2022; Wang et al., 2024). By utilizing these approaches, it becomes possible to depict the microbial trajectory during disease progression and identify individuals in the early stages of the disease (Fig. 4C). In addition to this, the development of micro-capsules that respond to intestinal pH value (Shalon et al., 2023) or external magnet field (Sun et al., 2024) may revolutionize the ways biospecimens are collected for early detection and personalized intervention in the future (Fig. 4D).

## Summary

The GI tract is a complex ecosystem with distinct gut microenvironments in each segment. These regions act as unique ecological niches with specific microbial composition and functions. The interactions between the host and microbiota influence the production of microbial metabolites, which play a vital role in regulating intestinal immune homeostasis and host metabolism. Understanding the pathophysiological significance of gut microbiota in different GI regions is crucial for developing targeted therapies.

Traditionally, fecal specimens have been used to assess gut and host metabolism. However, this approach does not fully capture the metabolic processes in the upper intestinal tract. The roles of microbial spatial organization and metabolic capacity in the small intestine have been overlooked in the understanding of metabolic diseases including obesity, DM, MASLD, IBD, and so on. Obtaining biological information from the small intestine is essential for unraveling host-microbiota interactions.

Each segment of the GI tract, with its resident gut microbiota, serves as a distinct functional ecological niche that regulates metabolic homeostasis. Disruption of the gut microenvironment can lead to the dislocation of gut microbiota along the GI tract, as seen in SIBO. This dislocation disrupts the balance between microbiota, metabolites, and the intestinal immune response, affecting metabolic homeostasis.

Conventional interventions like probiotics, prebiotics, surgery, FMT, and endoscopy-based procedures aim to correct the dislocation of gut microbiota, restoring the natural ecological niche and its functions. These interventions show promise in treating metabolic and GI disorders.

In the long term, prevention is prioritized over treatment. Integrating multidimensional microbial data, including oral and fecal samples, into AI-powered deep-learning prediction models can identify individuals at risk of developing diseases before clinical symptoms emerge. This approach holds potential for microbial data-driven precision medicine.

## Data Availability

Not applicable.

## References

[CIT0001] Abadie V , Sollid LM, Barreiro LB et al Integration of genetic and immunological insights into a model of celiac disease pathogenesis. Annu Rev Immunol 2011;29:493–525. 21219178 10.1146/annurev-immunol-040210-092915

[CIT0002] Adachi R , Honma Y, Masuno H et al Selective activation of vitamin D receptor by lithocholic acid acetate, a bile acid derivative. J Lipid Res 2005;46:46–57. 15489543 10.1194/jlr.M400294-JLR200

[CIT0003] Agus A , Planchais J, Sokol H. Gut microbiota regulation of tryptophan metabolism in health and disease. Cell Host Microbe 2018;23:716–724. 29902437 10.1016/j.chom.2018.05.003

[CIT0004] Ahlman H , Nilsson O. The gut as the largest endocrine organ in the body. Ann Oncol 2001;12 Suppl 2:S63–S68. 10.1093/annonc/12.suppl_2.s63 11762354

[CIT0005] Ahmad TR , Haeusler RA. Bile acids in glucose metabolism and insulin signalling — mechanisms and research needs. Nat Rev Endocrinol 2019;15:701–712. 31616073 10.1038/s41574-019-0266-7 PMC6918475

[CIT0006] Ahmed S , Macfarlane GT, Fite A et al Mucosa-associated bacterial diversity in relation to human terminal ileum and colonic biopsy samples. Appl Environ Microbiol 2007;73:7435–7442. 17890331 10.1128/AEM.01143-07 PMC2168195

[CIT0007] Albaugh VL , Banan B, Antoun J et al Role of bile acids and GLP-1 in mediating the metabolic improvements of bariatric surgery. Gastroenterology 2019;156:1041–1051.e4. 30445014 10.1053/j.gastro.2018.11.017 PMC6409186

[CIT0008] Allesøe RL , Lundgaard AT, Hernández Medina R et al Discovery of drug-omics associations in type 2 diabetes with generative deep-learning models. Nat Biotechnol 2023;41:399–408. 36593394 10.1038/s41587-022-01520-x PMC10017515

[CIT0009] An C , Chon H, Ku W et al Bile acids: major regulator of the gut microbiome. Microorganisms 2022;10:1792. 36144395 10.3390/microorganisms10091792 PMC9502002

[CIT0010] Angelakis E , Armougom F, Carrière F et al A metagenomic investigation of the duodenal microbiota reveals links with obesity. PLoS One 2015;10:e0137784. 26356733 10.1371/journal.pone.0137784 PMC4565581

[CIT0011] Atarashi K , Suda W, Luo C et al Ectopic colonization of oral bacteria in the intestine drives TH1 cell induction and inflammation. Science 2017;358:359–365. 29051379 10.1126/science.aan4526 PMC5682622

[CIT0012] Atreya R , Siegmund B. Location is important: differentiation between ileal and colonic Crohn’s disease. Nat Rev Gastroenterol Hepatol 2021;18:544–558. 33712743 10.1038/s41575-021-00424-6

[CIT0013] Baker JL , Mark Welch JL, Kauffman KM et al The oral microbiome: diversity, biogeography and human health. Nat Rev Microbiol 2024;22:89–104. 37700024 10.1038/s41579-023-00963-6 PMC11084736

[CIT0014] Barcik W , Wawrzyniak M, Akdis CA et al Immune regulation by histamine and histamine-secreting bacteria. Curr Opin Immunol 2017;48:108–113. 28923468 10.1016/j.coi.2017.08.011

[CIT0015] Blekhman R , Goodrich JK, Huang K et al Host genetic variation impacts microbiome composition across human body sites. Genome Biol 2015;16:191. 26374288 10.1186/s13059-015-0759-1 PMC4570153

[CIT0016] Bogatyrev SR , Rolando JC, Ismagilov RF. Self-reinoculation with fecal flora changes microbiota density and composition leading to an altered bile-acid profile in the mouse small intestine. Microbiome 2020;8:19. 32051033 10.1186/s40168-020-0785-4 PMC7017497

[CIT0017] Booijink CCGM , El-Aidy S, Rajilić-Stojanović M et al High temporal and inter-individual variation detected in the human ileal microbiota. Environ Microbiol 2010;12:3213–3227. 20626454 10.1111/j.1462-2920.2010.02294.x

[CIT0018] Buergel T , Steinfeldt J, Ruyoga G et al Metabolomic profiles predict individual multidisease outcomes. Nat Med 2022;28:2309–2320. 36138150 10.1038/s41591-022-01980-3 PMC9671812

[CIT0019] Bushyhead D , Quigley EMM. Small intestinal bacterial overgrowth-pathophysiology and its implications for definition and management. Gastroenterology 2022;163:593–607. 35398346 10.1053/j.gastro.2022.04.002

[CIT0020] Caminero A , Galipeau HJ, Mccarville JL et al Duodenal bacteria from patients with celiac disease and healthy subjects distinctly affect gluten breakdown and immunogenicity. Gastroenterology 2016;151:670–683. 27373514 10.1053/j.gastro.2016.06.041

[CIT0021] Canfora EE , Meex RCR, Venema K et al Gut microbial metabolites in obesity, NAFLD and T2DM. Nat Rev Endocrinol 2019;15:261–273. 30670819 10.1038/s41574-019-0156-z

[CIT0022] Cani PD , Amar J, Iglesias MA et al Metabolic endotoxemia initiates obesity and insulin resistance. Diabetes 2007;56:1761–1772. 17456850 10.2337/db06-1491

[CIT0023] Chaudhari SN , Harris DA, Aliakbarian H et al Bariatric surgery reveals a gut-restricted TGR5 agonist with anti-diabetic effects. Nat Chem Biol 2021;17:20–29. 32747812 10.1038/s41589-020-0604-z PMC7891870

[CIT0024] Chen B , Sun L, Zeng G et al Gut bacteria alleviate smoking-related NASH by degrading gut nicotine. Nature 2022;610:562–568. 36261549 10.1038/s41586-022-05299-4 PMC9589931

[CIT0025] Constante M , Libertucci J, Galipeau HJ et al Biogeographic variation and functional pathways of the gut microbiota in celiac disease. Gastroenterology 2022;163:1351–1363.e15. 35810781 10.1053/j.gastro.2022.06.088

[CIT0026] Dang JT , Mocanu V, Park H et al Ileal microbial shifts after Roux-en-Y gastric bypass orchestrate changes in glucose metabolism through modulation of bile acids and L-cell adaptation. Sci Rep 2021;11:23813. 34893681 10.1038/s41598-021-03396-4 PMC8664817

[CIT0027] Dantas Machado AC , Brown SD, Lingaraju A et al Diet and feeding pattern modulate diurnal dynamics of the ileal microbiome and transcriptome. Cell Reports 2022;40:111008. 35793637 10.1016/j.celrep.2022.111008 PMC9296000

[CIT0028] Darra A , Singh V, Jena A et al Hyperglycemia is associated with duodenal dysbiosis and altered duodenal microenvironment. Sci Rep 2023;13:11038. 37419941 10.1038/s41598-023-37720-x PMC10329043

[CIT0030] Debédat J , Le Roy T, Voland L et al The human gut microbiota contributes to type-2 diabetes non-resolution 5-years after Roux-en-Y gastric bypass. Gut Microbes 2022;14:2050635. 35435140 10.1080/19490976.2022.2050635 PMC9037437

[CIT0031] Devi TB , Devadas K, George M et al Low bifidobacterium abundance in the lower gut microbiota is associated with helicobacter pylori-related gastric ulcer and gastric cancer. Front Microbiol 2021;12:631140. 33717022 10.3389/fmicb.2021.631140 PMC7953064

[CIT0032] Devlin AS , Fischbach MA. A biosynthetic pathway for a prominent class of microbiota-derived bile acids. Nat Chem Biol 2015;11:685–690. 26192599 10.1038/nchembio.1864 PMC4543561

[CIT0033] Devlin AS , Marcobal A, Dodd D et al Modulation of a circulating uremic solute via rational genetic manipulation of the gut microbiota. Cell Host Microbe 2016;20:709–715. 27916477 10.1016/j.chom.2016.10.021 PMC5159218

[CIT0029] De Vos WM , Tilg H, Van Hul M et al Gut microbiome and health: mechanistic insights. Gut 2022;71:1020–1032. 35105664 10.1136/gutjnl-2021-326789 PMC8995832

[CIT0034] Donaldson GP , Lee SM, Mazmanian SK. Gut biogeography of the bacterial microbiota. Nat Rev Microbiol 2016;14:20–32. 26499895 10.1038/nrmicro3552 PMC4837114

[CIT0035] El Aidy S , Van den Bogert B, Kleerebezem M. The small intestine microbiota, nutritional modulation and relevance for health. Curr Opin Biotechnol 2015;32:14–20. 25308830 10.1016/j.copbio.2014.09.005

[CIT0036] El-Mir MY , Badia MD, Luengo N et al Increased levels of typically fetal bile acid species in patients with hepatocellular carcinoma. Clin Sci (Lond) 2001;100:499–508. 11294690

[CIT0037] Ermund A , Schütte A, Johansson MEV et al Studies of mucus in mouse stomach, small intestine, and colon. I. Gastrointestinal mucus layers have different properties depending on location as well as over the Peyer’s patches. Am J Physiol Gastrointest Liver Physiol 2013;305:G341–G347. 23832518 10.1152/ajpgi.00046.2013 PMC3761247

[CIT0038] Evans DF , Pye G, Bramley R et al Measurement of gastrointestinal pH profiles in normal ambulant human subjects. Gut 1988;29:1035–1041. 3410329 10.1136/gut.29.8.1035 PMC1433896

[CIT0039] Filardo S , Scalese G, Virili C et al The potential role of hypochlorhydria in the development of duodenal dysbiosis: a preliminary report. Front Cell Infect Microbiol 2022;12:854904. 35521214 10.3389/fcimb.2022.854904 PMC9062108

[CIT0040] Fiorucci S , Distrutti E, Carino A et al Bile acids and their receptors in metabolic disorders. Prog Lipid Res 2021;82:101094. 33636214 10.1016/j.plipres.2021.101094

[CIT0042] Flint HJ , Scott KP, Louis P et al The role of the gut microbiota in nutrition and health. Nat Rev Gastroenterol Hepatol 2012;9:577–589. 22945443 10.1038/nrgastro.2012.156

[CIT0041] Flint HJ , Duncan SH, Scott KP et al Links between diet, gut microbiota composition and gut metabolism. Proc Nutr Soc 2015;74:13–22. 25268552 10.1017/S0029665114001463

[CIT0043] Folz J , Culver RN, Morales JM et al Human metabolome variation along the upper intestinal tract. Nat Metab 2023;5:777–788. 37165176 10.1038/s42255-023-00777-z PMC10229427

[CIT0044] Fouladi F , Brooks AE, Fodor AA et al The role of the gut microbiota in sustained weight loss following Roux-en-Y gastric bypass surgery. Obes Surg 2019;29:1259–1267. 30604078 10.1007/s11695-018-03653-y

[CIT0045] Frazier K , Kambal A, Zale EA et al High-fat diet disrupts REG3γ and gut microbial rhythms promoting metabolic dysfunction. Cell Host Microbe 2022;30:809–823. 35439436 10.1016/j.chom.2022.03.030 PMC9281554

[CIT0046] Funabashi M , Grove TL, Wang M et al A metabolic pathway for bile acid dehydroxylation by the gut microbiome. Nature 2020;582:566–570. 32555455 10.1038/s41586-020-2396-4 PMC7319900

[CIT0047] Fung TC , Vuong HE, Luna CDG et al Intestinal serotonin and fluoxetine exposure modulate bacterial colonization in the gut. Nat Microbiol 2019;4:2064–2073. 31477894 10.1038/s41564-019-0540-4 PMC6879823

[CIT0048] Furuichi M , Kawaguchi T, Pust M-M et al Commensal consortia decolonize Enterobacteriaceae via ecological control. Nature 2024;633:878–886. 39294375 10.1038/s41586-024-07960-6 PMC11424487

[CIT0049] Gatarek P , Kaluzna-Czaplinska J. Trimethylamine N-oxide (TMAO) in human health. Excli J 2021;20:301–319. 33746664 10.17179/excli2020-3239 PMC7975634

[CIT0050] Gérard P. Metabolism of cholesterol and bile acids by the gut microbiota. Pathogens 2013;3:14–24. 25437605 10.3390/pathogens3010014 PMC4235735

[CIT0051] Ghosh S , Rubino F, Wismann P et al Westernized diet–induced insulin resistance in mice is associated with focal duodenal hyperplasia. Diabetes 2018;67:1900.

[CIT0052] Gniuli D , Calcagno A, Dalla Libera L et al High-fat feeding stimulates endocrine, glucose-dependent insulinotropic polypeptide (GIP)-expressing cell hyperplasia in the duodenum of Wistar rats. Diabetologia 2010;53:2233–2240. 20585935 10.1007/s00125-010-1830-9

[CIT0053] Gomari DP , Schweickart A, Cerchietti L et al Variational autoencoders learn transferrable representations of metabolomics data. Commun Biol 2022;5:645. 35773471 10.1038/s42003-022-03579-3 PMC9246987

[CIT0054] Gonzalez FJ , Jiang C, Patterson AD. An intestinal microbiota-farnesoid X receptor axis modulates metabolic disease. Gastroenterology 2016;151:845–859. 27639801 10.1053/j.gastro.2016.08.057 PMC5159222

[CIT0055] Gudan A , Jamioł-Milc D, Hawryłkowicz V et al The prevalence of small intestinal bacterial overgrowth in patients with non-alcoholic liver diseases: NAFLD, NASH, fibrosis, cirrhosis-A systematic review, meta-analysis and meta-regression. Nutrients 2022;14:5261. 36558421 10.3390/nu14245261 PMC9783356

[CIT0056] Hayashi H , Takahashi R, Nishi T et al Molecular analysis of jejunal, ileal, caecal and recto-sigmoidal human colonic microbiota using 16S rRNA gene libraries and terminal restriction fragment length polymorphism. J Med Microbiol 2005;54:1093–1101. 16192442 10.1099/jmm.0.45935-0

[CIT0057] Heinken A , Ravcheev DA, Baldini F et al Systematic assessment of secondary bile acid metabolism in gut microbes reveals distinct metabolic capabilities in inflammatory bowel disease. Microbiome 2019;7:75. 31092280 10.1186/s40168-019-0689-3 PMC6521386

[CIT0058] Hernández Medina R , Kutuzova S, Nielsen KN et al Machine learning and deep learning applications in microbiome research. ISME Commun 2022;2:98. 37938690 10.1038/s43705-022-00182-9 PMC9723725

[CIT0059] Hui W , Li T, Liu W et al Fecal microbiota transplantation for treatment of recurrent *C. difficile* infection: an updated randomized controlled trial meta-analysis. PLoS One 2019;14:e0210016. 30673716 10.1371/journal.pone.0210016 PMC6343888

[CIT0060] Hunt RH , Camilleri M, Crowe SE et al The stomach in health and disease. Gut 2015;64:1650–1668. 26342014 10.1136/gutjnl-2014-307595 PMC4835810

[CIT0061] Ianiro G , Maida M, Burisch J et al Efficacy of different faecal microbiota transplantation protocols for *Clostridium difficile* infection: a systematic review and meta-analysis. United Eur Gastroenterol J 2018;6:1232–1244. 10.1177/2050640618780762 PMC6169051 30288286

[CIT0062] Ierardi E , Losurdo G, Sorrentino C et al Macronutrient intakes in obese subjects with or without small intestinal bacterial overgrowth: an alimentary survey. Scand J Gastroenterol 2016;51:277–280. 26375876 10.3109/00365521.2015.1086020

[CIT0063] Imhann F , Vich Vila A, Bonder MJ et al Interplay of host genetics and gut microbiota underlying the onset and clinical presentation of inflammatory bowel disease. Gut 2018;67:108–119. 27802154 10.1136/gutjnl-2016-312135 PMC5699972

[CIT0064] Inagaki T , Choi M, Moschetta A et al Fibroblast growth factor 15 functions as an enterohepatic signal to regulate bile acid homeostasis. Cell Metab 2005;2:217–225. 16213224 10.1016/j.cmet.2005.09.001

[CIT0065] Ishizawa M , Akagi D, Makishima M. Lithocholic acid is a Vitamin D receptor ligand that acts preferentially in the ileum. Int J Mol Sci 2018;19:1975. 29986424 10.3390/ijms19071975 PMC6073204

[CIT0066] Jia W , Xie G, Jia W. Bile acid-microbiota crosstalk in gastrointestinal inflammation and carcinogenesis. Nat Rev Gastroenterol Hepatol 2018;15:111–128. 29018272 10.1038/nrgastro.2017.119 PMC5899973

[CIT0067] Jin D , Huang K, Xu M et al Deoxycholic acid induces gastric intestinal metaplasia by activating STAT3 signaling and disturbing gastric bile acids metabolism and microbiota. Gut Microbes2022a;14:2120744. 36067404 10.1080/19490976.2022.2120744 PMC9467587

[CIT0068] Jin W-B , Li T-T, Huo D et al Genetic manipulation of gut microbes enables single-gene interrogation in a complex microbiome. Cell2022b;185:547–562.e22. 35051369 10.1016/j.cell.2021.12.035 PMC8919858

[CIT0069] Kałużna-Czaplińska J , Gątarek P, Chirumbolo S et al How important is tryptophan in human health? Crit Rev Food Sci Nutr 2019;59:72–88. 28799778 10.1080/10408398.2017.1357534

[CIT0070] Kastl AJ , Terry NA, Wu GD et al The structure and function of the human small intestinal microbiota: current understanding and future directions. Cell Mol Gastroenterol Hepatol 2020;9:33–45. 31344510 10.1016/j.jcmgh.2019.07.006 PMC6881639

[CIT0071] Kaźmierczak-Siedlecka K , Daca A, Roviello G et al Interdisciplinary insights into the link between gut microbiome and gastric carcinogenesis-what is currently known? Gastric Cancer 2022;25:1–10. 34741681 10.1007/s10120-021-01260-y PMC8732854

[CIT0072] Kirwan JP , Courcoulas AP, Cummings DE et al Diabetes remission in the Alliance of Randomized Trials of Medicine Versus Metabolic Surgery in Type 2 Diabetes (ARMMS-T2D). Diabetes Care 2022;45:1574–1583. 35320365 10.2337/dc21-2441 PMC9490448

[CIT0073] Koh A , De Vadder F, Kovatcheva-Datchary P et al From dietary fiber to host physiology: short-chain fatty acids as key bacterial metabolites. Cell 2016;165:1332–1345. 27259147 10.1016/j.cell.2016.05.041

[CIT0074] Koh A , Molinaro A, Ståhlman M et al Microbially produced imidazole propionate impairs insulin signaling through mTORC1. Cell 2018;175:947–961.e17. 30401435 10.1016/j.cell.2018.09.055

[CIT0075] Kuang J , Wang J, Li Y et al Hyodeoxycholic acid alleviates non-alcoholic fatty liver disease through modulating the gut-liver axis. Cell Metab 2023;35:1752–1766.e8. 37591244 10.1016/j.cmet.2023.07.011

[CIT0076] Kunath BJ , Hickl O, Queirós P et al Alterations of oral microbiota and impact on the gut microbiome in type 1 diabetes mellitus revealed by integrated multi-omic analyses. Microbiome 2022;10:243. 36578059 10.1186/s40168-022-01435-4 PMC9795701

[CIT0077] Leite G , Pimentel M, Barlow GM et al Age and the aging process significantly alter the small bowel microbiome. Cell Reports 2021;36:109765. 34592155 10.1016/j.celrep.2021.109765

[CIT0078] Leite G , Rezaie A, Mathur R et al Defining small intestinal bacterial overgrowth by culture and high throughput sequencing. Clin Gastroenterol Hepatol 2023;22:259–270. 37315761 10.1016/j.cgh.2023.06.001

[CIT0079] Leonard MM , Valitutti F, Karathia H et al Microbiome signatures of progression toward celiac disease onset in at-risk children in a longitudinal prospective cohort study. Proc Natl Acad Sci USA 2021;118:e2020322118. 34253606 10.1073/pnas.2020322118 PMC8307711

[CIT0081] Li T , Guo H, Li H et al MicroRNA-92a-1-5p increases CDX2 by targeting FOXD1 in bile acids-induced gastric intestinal metaplasia. Gut 2019;68:1751–1763. 30635407 10.1136/gutjnl-2017-315318 PMC6839796

[CIT0080] Li N , Zuo B, Huang S et al Spatial heterogeneity of bacterial colonization across different gut segments following inter-species microbiota transplantation. Microbiome 2020;8:161. 33208178 10.1186/s40168-020-00917-7 PMC7677849

[CIT0082] Liou AP , Paziuk M, Luevano J-M et al Conserved shifts in the gut microbiota due to gastric bypass reduce host weight and adiposity. Sci Transl Med 2013;5:178ra41. 10.1126/scitranslmed.3005687 PMC3652229 23536013

[CIT0083] Liu C , Du M-X, Xie L-S et al Gut commensal Christensenella minuta modulates host metabolism via acylated secondary bile acids. Nat Microbiol 2024;9:434–450. 38233647 10.1038/s41564-023-01570-0

[CIT0084] Louis P , Hold GL, Flint HJ. The gut microbiota, bacterial metabolites and colorectal cancer. Nat Rev Microbiol 2014;12:661–672. 25198138 10.1038/nrmicro3344

[CIT0085] Lu R , Zhang Y-G, Xia Y et al Paneth cell alertness to pathogens maintained by Vitamin D receptors. Gastroenterology 2021;160:1269–1283. 33217447 10.1053/j.gastro.2020.11.015 PMC8808465

[CIT0086] Maccioni L , Gao B, Leclercq S et al Intestinal permeability, microbial translocation, changes in duodenal and fecal microbiota, and their associations with alcoholic liver disease progression in humans. Gut Microbes 2020;12:1782157. 32588725 10.1080/19490976.2020.1782157 PMC7524402

[CIT0087] Macfarlane S , Macfarlane GT. Regulation of short-chain fatty acid production. Proc Nutr Soc 2003;62:67–72. 12740060 10.1079/PNS2002207

[CIT0088] Madsen D , Beaver M, Chang L et al Analysis of bile acids in conventional and germfree rats. J Lipid Res 1976;17:107–111. 1270929

[CIT0089] Maini Rekdal V , Bess EN, Bisanz JE et al Discovery and inhibition of an interspecies gut bacterial pathway for Levodopa metabolism. Science 2019;364:eaau6323. 31196984 10.1126/science.aau6323 PMC7745125

[CIT0090] Makishima M , Lu TT, Xie W et al Vitamin D receptor as an intestinal bile acid sensor. Science 2002;296:1313–1316. 12016314 10.1126/science.1070477

[CIT0091] Makki K , Brolin H, Petersen N et al 6α-hydroxylated bile acids mediate TGR5 signalling to improve glucose metabolism upon dietary fiber supplementation in mice. Gut 2023;72:314–324. 35697422 10.1136/gutjnl-2021-326541 PMC9872241

[CIT0092] Marteau P , Pochart P, Doré J et al Comparative study of bacterial groups within the human cecal and fecal microbiota. Appl Environ Microbiol 2001; 67:4939–4942. 11571208 10.1128/AEM.67.10.4939-4942.2001 PMC93255

[CIT0093] Martinez-Guryn K , Hubert N, Frazier K et al Small intestine microbiota regulate host digestive and absorptive adaptive responses to dietary lipids. Cell Host Microbe 2018;23:458–469. 29649441 10.1016/j.chom.2018.03.011 PMC5912695

[CIT0094] Martinez-Guryn K , Leone V, Chang EB. Regional diversity of the gastrointestinal microbiome. Cell Host Microbe 2019;26:314–324. 31513770 10.1016/j.chom.2019.08.011 PMC6750279

[CIT0095] Mcgavigan AK , Garibay D, Henseler ZM et al TGR5 contributes to glucoregulatory improvements after vertical sleeve gastrectomy in mice. Gut 2017;66:226–234. 26511794 10.1136/gutjnl-2015-309871 PMC5512436

[CIT0096] Michel-Mata S , Wang X-W, Liu Y-Y et al Predicting microbiome compositions from species assemblages through deep learning. IMeta 2022;1:e3. 35757098 10.1002/imt2.3 PMC9221840

[CIT0097] Miele L , Valenza V, La Torre G et al Increased intestinal permeability and tight junction alterations in nonalcoholic fatty liver disease. Hepatology 2009;49:1877–1887. 19291785 10.1002/hep.22848

[CIT0098] Mingrone G , Panunzi S, De Gaetano A et al Metabolic surgery versus conventional medical therapy in patients with type 2 diabetes: 10-year follow-up of an open-label, single-centre, randomised controlled trial. Lancet 2021;397:293–304. 33485454 10.1016/S0140-6736(20)32649-0

[CIT0099] Mingrone G , van Baar AC, Devière J et al Safety and efficacy of hydrothermal duodenal mucosal resurfacing in patients with type 2 diabetes: the randomised, double-blind, sham-controlled, multicentre REVITA-2 feasibility trial. Gut 2022;71:254–264. 33597157 10.1136/gutjnl-2020-323608 PMC8761999

[CIT0100] Mohanty I , Allaband C, Mannochio-Russo H et al The changing metabolic landscape of bile acids - keys to metabolism and immune regulation. Nat Rev Gastroenterol Hepatol2024a;21:493–516. 38575682 10.1038/s41575-024-00914-3 PMC12248421

[CIT0101] Mohanty I , Mannochio-Russo H, Schweer JV et al The underappreciated diversity of bile acid modifications. Cell2024b;187:1801–1818.e20. 38471500 10.1016/j.cell.2024.02.019 PMC12248420

[CIT0102] Myronovych A , Bhattacharjee J, Salazar-Gonzalez R-M et al Assessment of the role of FGF15 in mediating the metabolic outcomes of murine Vertical Sleeve Gastrectomy (VSG). Am J Physiol Gastrointest Liver Physiol 2020;319:G669–G684. 32967428 10.1152/ajpgi.00175.2020 PMC7792670

[CIT0103] Nguyen NQ , Debreceni TL, Bambrick JE et al Accelerated intestinal glucose absorption in morbidly obese humans: relationship to glucose transporters, incretin hormones, and glycemia. J Clin Endocrinol Metab 2015;100:968–976. 25423571 10.1210/jc.2014-3144

[CIT0104] Nie Q , Luo X, Wang K et al Gut symbionts alleviate MASH through a secondary bile acid biosynthetic pathway. Cell 2024;187:2717–2734.e33. 38653239 10.1016/j.cell.2024.03.034

[CIT0105] Nissen JN , Johansen J, Allesøe RL et al Improved metagenome binning and assembly using deep variational autoencoders. Nat Biotechnol 2021;39:555–560. 33398153 10.1038/s41587-020-00777-4

[CIT0106] Noto JM , Piazuelo MB, Shah SC et al Iron deficiency linked to altered bile acid metabolism promotes Helicobacter pylori-induced inflammation-driven gastric carcinogenesis. J Clin Invest 2022;132:e147822. 35316215 10.1172/JCI147822 PMC9106351

[CIT0107] Patti M-E , Houten SM, Bianco AC et al Serum bile acids are higher in humans with prior gastric bypass: potential contribution to improved glucose and lipid metabolism. Obesity (Silver Spring) 2009;17:1671–1677. 19360006 10.1038/oby.2009.102 PMC4683159

[CIT0108] Peng X , Cheng L, You Y et al Oral microbiota in human systematic diseases. Int J Oral Sci 2022;14:14. 35236828 10.1038/s41368-022-00163-7 PMC8891310

[CIT0109] Pereira FC , Berry D. Microbial nutrient niches in the gut. Environ Microbiol 2017;19:1366–1378. 28035742 10.1111/1462-2920.13659 PMC5412925

[CIT0110] Peters TJ. Intestinal peptidases. Gut 1970;11:720–725. 4919261 10.1136/gut.11.8.720 PMC1553086

[CIT0111] Pols TWH , Noriega LG, Nomura M et al The bile acid membrane receptor TGR5 as an emerging target in metabolism and inflammation. J Hepatol 2011;54:1263–1272. 21145931 10.1016/j.jhep.2010.12.004 PMC3650458

[CIT0112] Porcari S , Benech N, Valles-Colomer M et al Key determinants of success in fecal microbiota transplantation: from microbiome to clinic. Cell Host Microbe 2023;31:712–733. 37167953 10.1016/j.chom.2023.03.020

[CIT0113] Quinn RA , Melnik AV, Vrbanac A et al Global chemical effects of the microbiome include new bile-acid conjugations. Nature 2020;579:123–129. 32103176 10.1038/s41586-020-2047-9 PMC7252668

[CIT0114] Quraishi MN , Widlak M, Bhala N et al Systematic review with meta-analysis: the efficacy of faecal microbiota transplantation for the treatment of recurrent and refractory Clostridium difficile infection. Aliment Pharmacol Ther 2017;46:479–493. 28707337 10.1111/apt.14201

[CIT0115] Rajilic-Stojanovic M , Figueiredo C, Smet A et al Systematic review: gastric microbiota in health and disease. Aliment Pharmacol Ther 2020;51:582–602. 32056247 10.1111/apt.15650

[CIT0116] Read E , Curtis MA, Neves JF. The role of oral bacteria in inflammatory bowel disease. Nat Rev Gastroenterol Hepatol 2021;18:731–742. 34400822 10.1038/s41575-021-00488-4

[CIT0117] Ridlon JM , Bajaj JS. The human gut sterolbiome: bile acid-microbiome endocrine aspects and therapeutics. Acta Pharm Sin B 2015;5:99–105. 26579434 10.1016/j.apsb.2015.01.006 PMC4629220

[CIT0118] Ridlon JM , Gaskins HR. Another renaissance for bile acid gastrointestinal microbiology. Nat Rev Gastroenterol Hepatol 2024;21:348–364. 38383804 10.1038/s41575-024-00896-2 PMC11558780

[CIT0119] Ridlon JM , Harris SC, Bhowmik S et al Consequences of bile salt biotransformations by intestinal bacteria. Gut Microbes2016a;7:22–39. 26939849 10.1080/19490976.2015.1127483 PMC4856454

[CIT0120] Ridlon JM , Wolf PG, Gaskins HR. Taurocholic acid metabolism by gut microbes and colon cancer. Gut Microbes2016b;7:201–215. 27003186 10.1080/19490976.2016.1150414 PMC4939921

[CIT0121] Rimal B , Collins SL, Tanes CE et al Bile salt hydrolase catalyses formation of amine-conjugated bile acids. Nature 2024;626:859–863. 38326609 10.1038/s41586-023-06990-w PMC10881385

[CIT0122] Roenneberg T , Merrow M. The circadian clock and human health. Curr Biol 2016;26:R432–R443. 27218855 10.1016/j.cub.2016.04.011

[CIT0123] Roland BC , Lee D, Miller LS et al Obesity increases the risk of small intestinal bacterial overgrowth (SIBO). Neurogastroenterol Motility 2018;30:e13199. 10.1111/nmo.13199 28940740

[CIT0124] Rubino F , Forgione A, Cummings DE et al The mechanism of diabetes control after gastrointestinal bypass surgery reveals a role of the proximal small intestine in the pathophysiology of type 2 diabetes. Ann Surg 2006;244:741–749. 17060767 10.1097/01.sla.0000224726.61448.1b PMC1856597

[CIT0125] Ruigrok RAAA , Collij V, Sureda P et al The composition and metabolic potential of the human small intestinal microbiota within the context of Inflammatory Bowel Disease. J Crohns Colitis 2021;15:1326–1338. 33515008 10.1093/ecco-jcc/jjab020 PMC8328293

[CIT0126] Ryan KK , Tremaroli V, Clemmensen C et al FXR is a molecular target for the effects of vertical sleeve gastrectomy. Nature 2014;509:183–188. 24670636 10.1038/nature13135 PMC4016120

[CIT0127] Ryder REJ , Laubner K, Benes M et al Endoscopic Duodenal-Jejunal bypass liner treatment for Type 2 diabetes and obesity: glycemic and cardiovascular disease risk factor improvements in 1,022 patients treated worldwide. Diabetes Care 2023;46:e89–e91. 36716004 10.2337/dc22-1952 PMC10090889

[CIT0128] Sato Y , Atarashi K, Plichta DR et al Novel bile acid biosynthetic pathways are enriched in the microbiome of centenarians. Nature 2021;599:458–464. 34325466 10.1038/s41586-021-03832-5

[CIT0129] Schauer PR , Bhatt DL, Kirwan JP et al Bariatric surgery versus intensive medical therapy for diabetes - 5-year outcomes. N Engl J Med 2017;376:641–651. 28199805 10.1056/NEJMoa1600869 PMC5451258

[CIT0130] Schugar RC , Gliniak CM, Osborn LJ et al Gut microbe-targeted choline trimethylamine lyase inhibition improves obesity via rewiring of host circadian rhythms. eLife 2022;11:e63998. 35072627 10.7554/eLife.63998 PMC8813054

[CIT0131] Seekatz AM , Schnizlein MK, Koenigsknecht MJ et al Spatial and temporal analysis of the stomach and small-intestinal microbiota in fasted healthy humans. MSphere 2019;4:e00126-19. 30867328 10.1128/mSphere.00126-19 PMC6416366

[CIT0132] Shalon D , Culver RN, Grembi JA et al Profiling the human intestinal environment under physiological conditions. Nature 2023;617:581–591. 37165188 10.1038/s41586-023-05989-7 PMC10191855

[CIT0134] Shanahan ER , Zhong L, Talley NJ et al Characterisation of the gastrointestinal mucosa-associated microbiota: a novel technique to prevent cross-contamination during endoscopic procedures. Aliment Pharmacol Ther 2016;43:1186–1196. 27086880 10.1111/apt.13622

[CIT0133] Shanahan ER , Kang S, Staudacher H et al Alterations to the duodenal microbiota are linked to gastric emptying and symptoms in functional dyspepsia. Gut 2023;72:929–938. 36167662 10.1136/gutjnl-2021-326158

[CIT0135] She J-J , Liu W-X, Ding X-M et al Defining the biogeographical map and potential bacterial translocation of microbiome in human ‘surface organs’. Nat Commun 2024;15:427. 38199995 10.1038/s41467-024-44720-6 PMC10781665

[CIT0136] Simon GL , Gorbach SL. Intestinal flora in health and disease. Gastroenterology 1984;86:174–193. 6357937

[CIT0137] Skrzydło-Radomańska B , Cukrowska B. How to recognize and treat small intestinal bacterial overgrowth? J Clin Med 2022;11:6017. 36294338 10.3390/jcm11206017 PMC9604644

[CIT0138] Sommer F , Bäckhed F. The gut microbiota--masters of host development and physiology. Nat Rev Microbiol 2013;11:227–238. 23435359 10.1038/nrmicro2974

[CIT0140] Song X , Sun X, Oh SF et al Microbial bile acid metabolites modulate gut RORγ^+^ regulatory T cell homeostasis. Nature 2020;577:410–415. 31875848 10.1038/s41586-019-1865-0 PMC7274525

[CIT0139] Song I , Gotoh Y, Ogura Y et al Comparative genomic and physiological analysis against *Clostridium scindens* reveals Eubacterium sp. c-25 as an atypical deoxycholic acid producer of the human gut microbiota. Microorganisms 2021;9:2254. 34835380 10.3390/microorganisms9112254 PMC8623032

[CIT0141] Sorbara MT , Pamer EG. Microbiome-based therapeutics. Nat Rev Microbiol 2022;20:365–380. 34992261 10.1038/s41579-021-00667-9

[CIT0142] Sroka-Oleksiak A , Młodzińska A, Bulanda M et al Metagenomic analysis of duodenal microbiota reveals a potential biomarker of dysbiosis in the course of obesity and type 2 diabetes: a pilot study. J Clin Med 2020;9:369. 32013181 10.3390/jcm9020369 PMC7074165

[CIT0143] Strathdee SA , Hatfull GF, Mutalik VK et al Phage therapy: from biological mechanisms to future directions. Cell 2023;186:17–31. 36608652 10.1016/j.cell.2022.11.017 PMC9827498

[CIT0144] Subramaniam S , Fletcher C. Trimethylamine N-oxide: breathe new life. Br J Pharmacol 2018;175:1344–1353. 28745401 10.1111/bph.13959 PMC5866995

[CIT0146] Sun M , Wu W, Liu Z et al Microbiota metabolite short chain fatty acids, GPCR, and inflammatory bowel diseases. J Gastroenterol 2017;52:1–8. 27448578 10.1007/s00535-016-1242-9 PMC5215992

[CIT0145] Sun L , Cai J, Gonzalez FJ. The role of farnesoid X receptor in metabolic diseases, and gastrointestinal and liver cancer. Nat Rev Gastroenterol Hepatol 2021;18:335–347. 33568795 10.1038/s41575-020-00404-2

[CIT0147] Sun Y , Zhang W, Gu J et al Magnetically driven capsules with multimodal response and multifunctionality for biomedical applications. Nat Commun 2024;15:1839. 38424039 10.1038/s41467-024-46046-9 PMC10904804

[CIT0148] Takahashi JS. Transcriptional architecture of the mammalian circadian clock. Nat Rev Genet 2017;18:164–179. 27990019 10.1038/nrg.2016.150 PMC5501165

[CIT0150] Thaiss CA , Zeevi D, Levy M et al A day in the life of the meta-organism: diurnal rhythms of the intestinal microbiome and its host. Gut Microbes 2015;6:137–142. 25901892 10.1080/19490976.2015.1016690 PMC4615721

[CIT0149] Thaiss CA , Levy M, Korem T et al Microbiota diurnal rhythmicity programs host transcriptome oscillations. Cell 2016;167:1495–1510.e12. 27912059 10.1016/j.cell.2016.11.003

[CIT0151] Tolhurst G , Heffron H, Lam YS et al Short-chain fatty acids stimulate glucagon-like peptide-1 secretion via the G-protein-coupled receptor FFAR2. Diabetes 2012;61:364–371. 22190648 10.2337/db11-1019 PMC3266401

[CIT0152] Tomioka S , Seki N, Sugiura Y et al Cooperative action of gut-microbiota-accessible carbohydrates improves host metabolic function. Cell Reports 2022;40:111087. 35858544 10.1016/j.celrep.2022.111087

[CIT0153] Treherne JE. Gut absorption. Annu Rev Entomol 1967;12:43–58. 5340725 10.1146/annurev.en.12.010167.000355

[CIT0154] Tropini C , Earle KA, Huang KC et al The gut microbiome: connecting spatial organization to function. Cell Host Microbe 2017;21:433–442. 28407481 10.1016/j.chom.2017.03.010 PMC5576359

[CIT0155] Tuganbaev T , Mor U, Bashiardes S et al Diet diurnally regulates small intestinal microbiome-epithelial-immune homeostasis and enteritis. Cell 2020;182:1441–1459.e21. 32888430 10.1016/j.cell.2020.08.027

[CIT0157] van Baar ACG , Holleman F, Crenier L et al Endoscopic duodenal mucosal resurfacing for the treatment of type 2 diabetes mellitus: one year results from the first international, open-label, prospective, multicentre study. Gut 2020;69:295–303. 31331994 10.1136/gutjnl-2019-318349 PMC6984054

[CIT0158] van Baar ACG , Meiring S, Holleman F et al Alternative treatments for type 2 diabetes and associated metabolic diseases: medical therapy or endoscopic duodenal mucosal remodelling? Gut 2021;70:2196–2204. 34497147 10.1136/gutjnl-2020-323931 PMC8515106

[CIT0156] van Baar ACG , Devière J, Hopkins D et al Durable metabolic improvements 2 years after duodenal mucosal resurfacing (DMR) in patients with type 2 diabetes (REVITA-1 Study). Diabetes Res Clin Pract 2022;184:109194. 35032562 10.1016/j.diabres.2022.109194

[CIT0159] van Kessel SP , Frye AK, El-Gendy AO et al Gut bacterial tyrosine decarboxylases restrict levels of levodopa in the treatment of Parkinson’s disease. Nat Commun 2019;10:310. 30659181 10.1038/s41467-019-08294-y PMC6338741

[CIT0160] Villmones HC , Svanevik M, Ulvestad E et al Investigating the human jejunal microbiota. Sci Rep 2022;12:1682. 35102222 10.1038/s41598-022-05723-9 PMC8803847

[CIT0161] Vonaesch P , Araújo JR, Gody J-C et al Stunted children display ectopic small intestinal colonization by oral bacteria, which cause lipid malabsorption in experimental models. Proc Natl Acad Sci USA 2022;119:e2209589119. 36197997 10.1073/pnas.2209589119 PMC9573096

[CIT0162] Vujkovic-Cvijin I , Dunham RM, Iwai S et al Dysbiosis of the gut microbiota is associated with HIV disease progression and tryptophan catabolism. Sci Transl Med 2013;5:193–ra91. 10.1126/scitranslmed.3006438 PMC4094294 23843452

[CIT0163] Wahlström A , Sayin SI, Marschall HU et al Intestinal crosstalk between bile acids and microbiota and its impact on host metabolism. Cell Metab 2016;24:41–50. 27320064 10.1016/j.cmet.2016.05.005

[CIT0164] Wang M , Ahrné S, Jeppsson B et al Comparison of bacterial diversity along the human intestinal tract by direct cloning and sequencing of 16S rRNA genes. FEMS Microbiol Ecol 2005;54:219–231. 16332321 10.1016/j.femsec.2005.03.012

[CIT0165] Wang S , Kuang J, Zhang H et al Bile acid-microbiome interaction promotes gastric carcinogenesis. Adv Sci 2022;9:e2200263. 10.1002/advs.202200263 PMC9165488 35285172

[CIT0166] Wang X-W , Sun Z, Jia H et al Identifying keystone species in microbial communities using deep learning. Nat Ecol Evol 2024;8:22–31. 37974003 10.1038/s41559-023-02250-2 PMC12125608

[CIT0167] Williams BB , van Benschoten AH, Cimermancic P et al Discovery and characterization of gut microbiota decarboxylases that can produce the neurotransmitter tryptamine. Cell Host Microbe 2014;16:495–503. 25263219 10.1016/j.chom.2014.09.001 PMC4260654

[CIT0168] Wu Q , Liang X, Wang K et al Intestinal hypoxia-inducible factor 2α regulates lactate levels to shape the gut microbiome and alter thermogenesis. Cell Metab 2021;33:1988–2003.e7. 34329568 10.1016/j.cmet.2021.07.007

[CIT0169] Xu S-S , Wang N, Huang L et al Changes in the mucosa-associated microbiome and transcriptome across gut segments are associated with obesity in a Metabolic Syndrome Porcine Model. Microbiol Spectr 2022;10:e0071722. 35862956 10.1128/spectrum.00717-22 PMC9430857

[CIT0170] Yadav J , Liang T, Qin T et al Gut microbiome modified by bariatric surgery improves insulin sensitivity and correlates with increased brown fat activity and energy expenditure. Cell Rep Med 2023;4:101051. 37196633 10.1016/j.xcrm.2023.101051 PMC10213984

[CIT0172] Yang I , Nell S, Suerbaum S. Survival in hostile territory: the microbiota of the stomach. FEMS Microbiol Rev 2013;37:736–761. 23790154 10.1111/1574-6976.12027

[CIT0171] Yang G , Hong E, Oh S et al Non-viable *Lactobacillus johnsonii* JNU3402 protects against diet-induced obesity. Foods 2020;9:1494. 33086627 10.3390/foods9101494 PMC7603363

[CIT0173] Yang J , He Q, Lu F et al A distinct microbiota signature precedes the clinical diagnosis of hepatocellular carcinoma. Gut Microbes 2023;15:2201159. 37089022 10.1080/19490976.2023.2201159 PMC10128432

[CIT0174] Yano JM , Yu K, Donaldson GP et al Indigenous bacteria from the gut microbiota regulate host serotonin biosynthesis. Cell 2015;161:264–276. 25860609 10.1016/j.cell.2015.02.047 PMC4393509

[CIT0175] Yilmaz B , Fuhrer T, Morgenthaler D et al Plasticity of the adult human small intestinal stoma microbiota. Cell Host Microbe 2022;30:1773–1787. 36318918 10.1016/j.chom.2022.10.002

[CIT0176] Zhang X , Chen G, Zhang H et al Bioinspired oral delivery devices. Nat Rev Bioeng 2023;1:208–225.

[CIT0177] Zhao L , Zhang F, Ding X et al Gut bacteria selectively promoted by dietary fibers alleviate type 2 diabetes. Science (New York, N.Y.) 2018;359:1151–1156. 29590046 10.1126/science.aao5774

[CIT0179] Zheng X , Huang F, Zhao A et al Bile acid is a significant host factor shaping the gut microbiome of diet-induced obese mice. BMC Biol 2017;15:120. 29241453 10.1186/s12915-017-0462-7 PMC5731064

[CIT0178] Zheng X , Chen T, Jiang R et al Hyocholic acid species improve glucose homeostasis through a distinct TGR5 and FXR signaling mechanism. Cell Metab 2021;33:791–803. 33338411 10.1016/j.cmet.2020.11.017

[CIT0180] Zhu L , Fu J, Xiao X et al Faecal microbiota transplantation-mediated jejunal microbiota changes halt high-fat diet-induced obesity in mice via retarding intestinal fat absorption. Microb Biotechnol 2022;15:337–352. 34704376 10.1111/1751-7915.13951 PMC8719817

[CIT0181] Zmora N , Zilberman-Schapira G, Suez J et al Personalized gut mucosal colonization resistance to empiric probiotics is associated with unique host and microbiome features. Cell 2018;174:1388–1405. 30193112 10.1016/j.cell.2018.08.041

